# Tailoring topological edge states with photonic crystal nanobeam cavities

**DOI:** 10.1038/s41598-020-79915-6

**Published:** 2021-01-13

**Authors:** Yongkang Gong, Liang Guo, Stephan Wong, Anthony J. Bennett, Sang Soon Oh

**Affiliations:** 1 School of Physics and Astronomy, Cardiff University, Cardiff, CF24 3AA UK; 2 Department of Basic Science, Jilin Jianzhu University, 5088 Xincheng Street, Changchun, 130118 China; 3 School of Engineering, Cardiff University, Cardiff, CF24 3AA UK

**Keywords:** Engineering, Optics and photonics

## Abstract

The realization of topological edge states (TESs) in photonic systems has provided unprecedented opportunities for manipulating light in novel manners. The Su–Schrieffer–Heeger (SSH) model has recently gained significant attention and has been exploited in a wide range of photonic platforms to create TESs. We develop a photonic topological insulator strategy based on SSH photonic crystal nanobeam cavities. In contrast to the conventional photonic SSH schemes which are based on alternately tuned coupling strength in one-dimensional lattice, our proposal provides higher flexibility and allows tailoring TESs by manipulating mode coupling in a two-dimensional manner. We reveal that the proposed hole-array based nanobeams in a dielectric membrane can selectively tailor single or double TESs in the telecommunication region by controlling the coupling strength of the adjacent SSH nanobeams in both transverse and axial directions. Our finding provides an additional degree of freedom in exploiting the SSH model for integrated topological photonic devices and functionalities based on the well-established photonic crystal nanobeam cavity platforms.

## Introduction

In comparison with traditional photonic defect states that are sensitive to perturbations, edge states from photonic topological insulators (PTIs) are robust against local perturbations and immune to back scattering. This leads to intriguing and unexpected photonic devices and functionalities for electromagnetic wave manipulations, such as unidirectional light and backscattering-free light transport^1–3^, topological lasing^4–11^, light steering^12^, nonlinear parametric generation^13,14^, protection of single photons^15^, and entangled photonic states^16,17^. The generation of topological edge states (TESs), which are the core of the emerging field of photonic PTIs, has recently made remarkable progress and has inspired various fundamentally different topological approaches. For example, a photonic analogue of a quantum Hall topological insulator was developed in the microwave regime using gyromagnetic materials with a strong magnetic field applied to break the time-reversal symmetry, and unidirectional backscattering-immune TESs were observed^18^. Later, a number of proposals have been put forward to realize TESs free of external magnetic fields by temporal modulation of photonic crystals to mimic time-reversal-symmetry breaking^19–22^. All-dielectric PTI approaches based on pseudo-time-reversal symmetry^15,23–28^ and valley Hall photonic crystals with broken spatial-inversion symmetry^2,3,29–37^ have proven to be effective in generating TESs at the subwavelength scale. Another elegant and powerful subwavelength-scale nontrivial topology approach is the Su–Schrieffer–Heeger (SSH) model, which was originally introduced to describe fractionalized electric charges in polyacetylene and has recently attracted considerable attention in photonics. Photonic SSH structures have been extensively investigated and applied to a broad range of platforms from microwave to optical regime including photonic superlattices^38^, plasmonic waveguides^39^, zigzag arrays of dielectric resonator chain^40^, dielectric nanoparticles^41^, polariton micropillars^7^, micro-ring resonator arrays^42,43^, photonic crystal L3 nanocavity dimer array^44^, dielectric waveguides^45–49^. These platforms open avenues to on-chip photonic devices for robust topologically protected light manipulation.

In this paper, we propose a new SSH scheme based on photonic crystal (PhC) nanobeam cavities and demonstrate that TESs can be generated by controlling the coupling strength of the nanobeams in two dimensions, which differs from the reported photonic SSH structures that utilize alternate modulation of the coupling strength in a one-dimensional lattice. The SSH nanobeams can allow two types of TESs in the telecommunication wavelength region, and more importantly the TESs can be selectively enabled by engineering the transverse spacing and axial shift between the adjacent nanobeams in the SSH structures. PhC nanobeam cavities are well-established platforms due to their exceptional cavity figures of merit (ultra-high Q factor and ultra-small mode volume)^50,51^, small footprint, excellent complementary metal–oxide–semiconductor (CMOS) compatible properties, and have found tremendous photonic integrated circuit applications such as sensors^52^, nanolasers^53,54^, optical switches^55^, electro-optical modulators^56^ and single-photon sources^57–59^ in optical systems, nonlinear mixing^60^ and wavelength conversion^61^ in opto-mechanical systems, and thermal management in opto-thermal systems^62,63^. We believe our work could pave a new avenue to exploit edge states in the well-established PhC nanobeam cavity platforms for various passive and active integrated topological devices.

## Results

### Concept, implementation, and analysis of the SSH nanobeams

**Figure 1:**
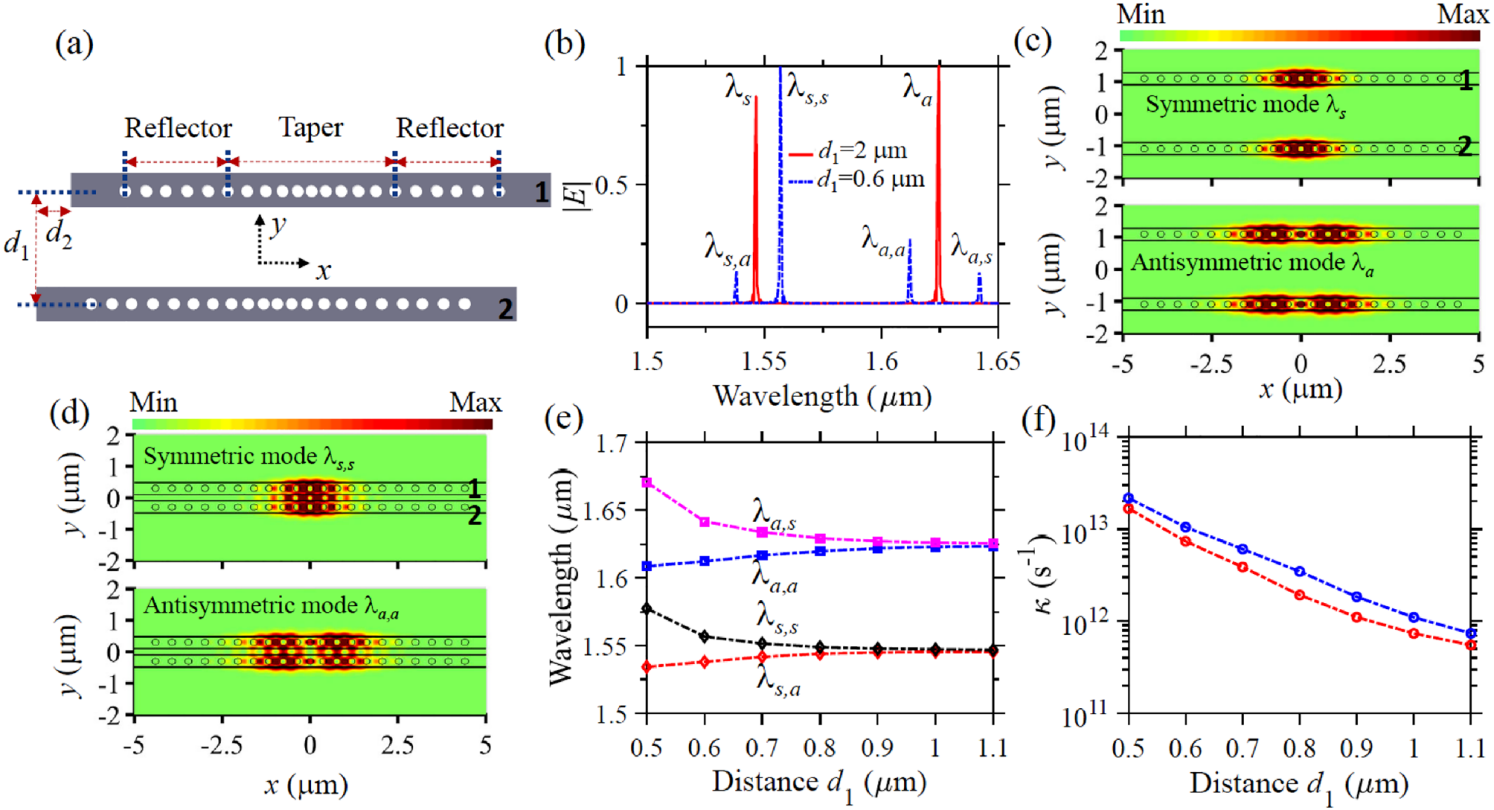
Wavelength splitting characteristics of the resonance modes of the two coupled PhC nanobeams. (**a**) Schematic top view of the two identical nanobeams with transverse spacing $d_{1}$ and axial shift $d_{2}$, where each nanobeam consists of a linear array of air holes with the same radius of $r$ in a semiconductor membrane with thickness $t$ and width $w$. (**b**) The normalized $\left| {\mathbf{E}} \right|$ spectra of the nanobeams when $d_{1} = 2\; {\upmu{\text{m}}}$ and $d_{1} = 0.6\; {\upmu{\text{m}}}$, showing the appearance of the wavelength splitting phenomenon at small $d_{1}$ due to strong mode coupling. (**c**) The electric field distribution $\left| {\mathbf{E}} \right|$ of the symmetric mode at $\lambda_{s} = 1.546\; {\upmu{\text{ m}}}$ and the antisymmetric mode at $\lambda_{a} = 1.624 \; {\upmu{\text{m}}}$ in the plane of the central membrane (i.e., the $z = 0\;\upmu{\text{m}}$ plane) when $d_{1} = 2\; {\upmu{\text{m}}}$, respectively. (**d**) The field distribution $\left| {\mathbf{E}} \right|$ of the symmetric mode at $\lambda_{s,s} = 1.557\; {\upmu{\text{m}}}$ and the antisymmetric mode at $\lambda_{a,a} = 1.612\; {\upmu{\text{m}}}$ when $d_{1} = 0.6 \; {\upmu{\text{m}}}$, respectively. The field distribution $H_{z}$ of these modes are given in Figs. S1 and S2 in Supplementary Information. (**e**) Evolution of the four splitting wavelengths (i.e., $\lambda_{s,a}$, $\lambda_{s,s}$, $\lambda_{a,a}$, and $\lambda_{a,s}$) to the change of $d_{1}$. (**f**) Dependence of the coupling strength $\kappa$ of the first (red curve) and the second resonance modes (blue curve) on $d_{1}$. In above simulations, each nanobeam has six air holes with hole-to-hole spacing of $0.47\; {\upmu{\text{m}}}$ in the reflector sections, and has nine nanoholes in the taper section with the hole-to-hole spacing decreased with a step of 20 nm from either side of the nanobeam to its center. The other geometrical parameters are $t = 0.3\; {\upmu{\text{m}}}$, $w = 0.4\; {\upmu{\text{m}}}$*, *$r = 0.1\; {\upmu{\text{m}}}$*,* and $d_{2} = 0 \;{\upmu{\text{m}}}$. The refractive index of the semiconductor membrane is 3.3.

Our proposed SSH scheme utilizes a free-standing PhC nanobeam array with each array consisting of a row of air holes in a semiconductor membrane with thickness $t$ and width $w$. Since the topological property of the proposed SSH nanobeam structure arises from the alternating coupling strength between the adjacent nanobeams, we first investigate the optical coupling characteristic of two identical nanobeams, as schematically depicted in Fig. 1a, where the two nanobeams have transverse spacing of $d_{1}$ and axial shift of $d_{2}$ with each nanobeam incorporating six air holes in the reflector sections and nine air holes in the taper section. All the holes have the same diameter. The hole-to-hole spacing in the two reflector sections is the same but reduces gradually from both sides to the center of the taper section to form an optical cavity. We perform designs and analyses by three-dimensional (3D) finite-difference time-domain (FDTD) method^64^. The obtained results demonstrate that when the two nanobeams have a large transverse spacing such as $d_{1} = 2 \; \upmu{\text{m}}$, each nanobeam generates two resonance modes in the telecommunication region and the resonance modes in the two nanobeams do not couple to each other, as illustrated by the electric field distribution |*E*| in Fig. 1c. We note from the optical spectrum in Fig. 1b and the field distribution $H_{z}$ in Fig. S1 in Supplementary Information that the first resonance mode at $\lambda_{s } = 1.546\; \upmu{\text{m}}$ has symmetric ${ }H_{z}$ field distribution with respect to the $x = 0\; \upmu{\text{m}}$ plane, while the second resonance mode at $\lambda_{a } = 1.624\; \upmu{\text{m}}$ has antisymmetric ${ }H_{z}$ field profile with respect to the same plane. The symmetric and antisymmetric modes have Q factor of $7.1 \times 10^{4}$ and $1.1 \times 10^{4}$, and mode volume $V_{{{\text{eff}}}}$ of $0.5\left( {{\uplambda }/n} \right)^{3} { }$ and $0.9\left( {{\uplambda }/n} \right)^{3}$, respectively, where $\lambda$ is the resonance mode wavelength and $n$ is the refractive index of the semiconductor membrane. The mode volume is calculated by $V_{\text{eff}} = \frac{{\mathop \smallint \nolimits_{V}^{{}} \varepsilon \left( {\mathbf{r}} \right)\left| {\mathbf{E}\left( {\mathbf{r}} \right)} \right|^{2} dr^{3} }}{{{\text{max}}\left[ {\varepsilon \left( {\mathbf{r}} \right)\left| {\mathbf{E}\left( {\mathbf{r}} \right)} \right|^{2} } \right]}}$^65^, where $\varepsilon \left( r \right)$, $\left| {\mathbf{E}\left( \mathbf{r} \right)} \right|$, and $V$ are the dielectric constant, the electric field strength, and the volume of the nanobeam cavity, respectively. We stress that the Q factor and the mode volume can be further improved by optimizing the hole diameters, the number of holes, and the hole-to-hole spacings as reported in Refs.^51,66,67^. Whilst the two resonance modes in each nanobeam do not couple to each other because they are separated at large transverse spacing of $d_{1} = 2\; \upmu{\text{m}}$ (Fig. 1c), they start to couple when $d_{1}$ reduces to such as $d_{1} = 0.6\; \upmu{\text{m}}$ (Fig. 1d). As a result, wavelength splitting of the two resonance modes occurs. We see from the optical spectra (Fig. 1b) and the field distribution $H_{z}$ (Fig. S2 in Supplementary Information) that the first resonance mode at $\lambda_{s}$, which is symmetric with respect to reflection by the $x = 0$ plane, is split into antisymmetric and symmetric modes with respect to reflection by the $y = 0 \; \upmu{\text{m}}$ plane. In the optical spectrum, the antisymmetric (symmetric) mode is located at shorter (longer) wavelength of $\lambda_{s,a }$ ($\lambda_{s,s}$). A similar phenomenon happens to the second resonance mode at $\lambda_{a }$ which is split into an antisymmetric mode with respect to the reflection by the $y = 0 \; \upmu{\text{m}}$ plane at shorter wavelength of $\lambda_{a,a }$ and a symmetric mode with respect to the reflection by the $y = 0 \; \upmu{\text{m}}$ at longer wavelength of $\lambda_{a,s}$. The wavelength splitting strongly depends on the transverse spacing $d_{1}$ (Fig. 1e) and increases when $d_{1}$ decreases due to the presence of stronger mode coupling. We derive the coupling strength $\kappa$ of the two resonance modes by $\kappa = \frac{\pi c\Delta \lambda }{{\lambda^{2} }}$^68^, where $c$ is the speed of light in vacuum, and $\Delta \lambda = |\lambda_{s,a} - \lambda_{s,s} |$ ($\Delta \lambda = \left| {\lambda_{a,a} - \lambda_{a,s} } \right|$) and $\lambda = \lambda_{s }$ ($\lambda = \lambda_{a }$) is the splitting wavelength difference and the wavelength of the first (second) resonance mode, respectively. Figure 1f shows that the first resonance mode has lower coupling strength than that of the second resonance mode under the same $d_{1}$ due to smaller mode volume.

**Figure 2:**
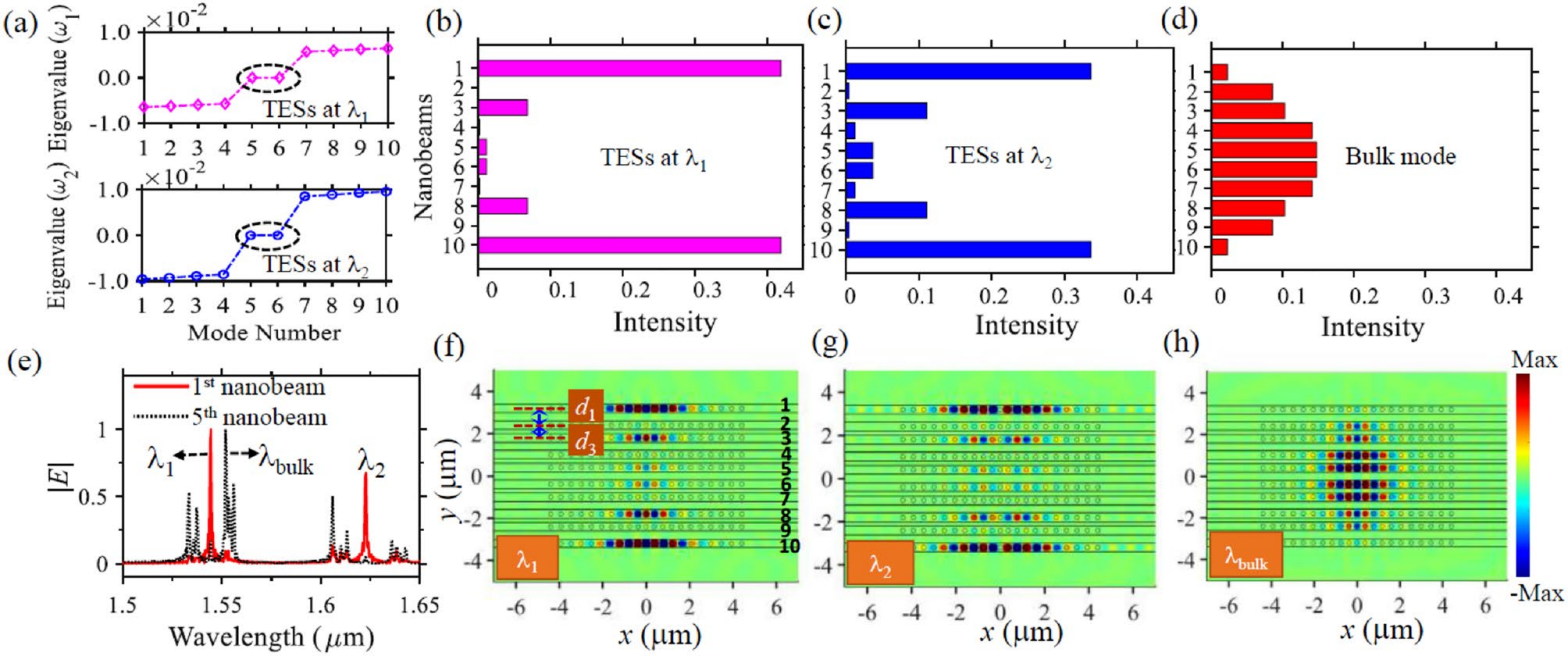
Generation of TESs with the finite SSH nanobeams implemented by the tight-binding model (**a**–**d**) and the 3D FDTD method (**e**–**g**). (**a**) The normalized eigenvalues of the first TES (upper) and the second TES (lower), indicating the existence of the two degenerated zero-energy TESs at both the first resonance wavelength $\lambda_{1}$ and the second resonance wavelength $\lambda_{2}$. $\omega_{1}$ and $\omega_{2}$ are the angular frequency of the two TESs with respect to $\lambda_{1}$ and $\lambda_{2}$ respectively. The field distribution (i.e., the real part of the eigenvectors) of these TESs have localized field at edge nanobeams in symmetric or antisymmetric manners, as plotted in Fig. S4 in Supplementary Information. (**b**–**d**) The conventionally normalized intensity (i.e., the absolute value of the eigenvector) of the TESs at $\lambda_{1}$, $\lambda_{2}$, and one of the bulk modes, respectively. (**e**) The normalized $\left| E \right|$ spectra of the edge nanobeam (i.e., the 1st nanobeam) and the middle nanobeam (i.e., the 5th nanobeam) of the SSH structure. The first (fifth) nanobeam has the same spectra to that of the tenth (sixth) nanobeam due to structure symmetry. (**f**–**h**) The real part of the field distribution $H_{z}$ of the first TES at $\lambda_{1} = 1.546 \;{\upmu{\text{m}}}$, the second TES at $\lambda_{2} = 1.624 \;{\upmu{\text{m}}}$, and the bulk mode at $\lambda_{\text{bulk}} = 1.552 \;{\upmu{\text{m}}}$ in the middle plane of the nanobeam membrane, respectively. The SSH structures under study have 10 identical nanobeams with alternative neighboring distance of $d_{1} = 0.8 \;{\upmu{\text{m}}}$ and $d_{3} = 0.6 \;{\upmu{\text{m}}}$, and zero axial shift (i.e., $d_{2} = 0 \;{\upmu{\text{m}}}$), as denoted by the structure outline in (**f**). Each nanobeam of the SSH structure has geometrical parameters same as those in Fig. 1.

Based on the property of the optical coupling strength varying with the nanobeams’ transverse spacing, we construct finite SSH nanobeams to generate TESs by alternately changing the transverse spacing between successive nanobeams. For the examples considered here we use structures with 10 nanobeams, but the results have general applicability to other even-number nanobeam arrays. When our SSH structures have an odd number of nanobeams, the generated TESs have the similar physical mechanism and optical properties (although there is a difference that odd number of SSH nanobeams allows a single zero-energy mode while an even number of nanobeams generates two degenerate zero-energy modes^69^). The developed SSH structure involves a nontrivial termination with spacing of $d_{1} = 0.8 \;{\upmu{\text{m}}}$ and $d_{3} = 0.6 \;{\upmu{\text{m}}}$ (as denoted by the structure outline in Fig. 2f), which gives rise to alternative intra-coupling of $\kappa_{1} = 5.5 \times 10^{11} {\text{s}}^{ - 1}$ and inter-coupling of $\kappa_{2} = 7.3 \times 10^{12} {\text{s}}^{ - 1}$ for the first resonance mode, and alternative intra-coupling of $\kappa_{1} = 7.4 \times 10^{11} {\text{s}}^{ - 1}$ and inter-coupling of $\kappa_{2} = 1.1 \times 10^{13} {\text{s}}^{ - 1}$ for the second resonance mode. The winding number determines the topological invariants of the SSH nanobeam systems. The winding number is 0 for the trivial topological phase when the intra-coupling is larger than the inter-coupling and is 1 for the nontrivial topological phase that TESs are introduced when the intra-coupling is smaller than the inter-coupling^44^. We perform tight binding analysis by incorporating the above coupling strength into the Hamiltonian of the SSH system (see Eq. (S1) in Supplementary Information) to obtain the eigenvalues and the eigenvectors. Figure 2a demonstrates that the SSH configuration allows two degenerate zero-energy modes at both the first resonance wavelength $\lambda_{1 }$ and the second resonance wavelength $\lambda_{2 }$. At each of the resonance wavelengths, one zero-energy mode has symmetric localized field at the edge nanobeams (i.e., the first and tenth nanobeams), while the other zero-energy mode has an asymmetric localized field profile (Fig. S4 in Supplementary Information). The intensity of the TESs at $\lambda_{1 }$ and $\lambda_{2 }$ are mainly localized at the center of the edge nanobeams and decay exponentially to the middle nanobeams, as depicted in Fig. 2b,c. Conversely, bulk modes do not have localized fields in the edge nanobeams and have fields mainly distributed in the middle nanobeams (Fig. 2d). Having two TESs simultaneously in one photonic system could allow us to study nonlinear interaction of the edge modes at two different wavelengths. For example, we could actively control of one edge mode with the other one assisted by optical nonlinearity with potential applications of all-optical switching, cross-phase modulation and four-wave mixing etc. We validate the tight binding analysis by implementing 3D FDTD modellings. Figure 2e demonstrates that the first nanobeam supports two strong spectral peaks at wavelengths of $\lambda_{1 } = 1.546\;{\upmu{\text{m}}}$ and $\lambda_{2 } = 1.642\;\upmu{\text{m}}$, which corresponds to the two TESs of the SSH structures with electric field mainly localized at the first and tenth nanobeams (Fig. 2f,g). We note that from Fig. 2e that the fifth nanobeam supports multiple spectral peaks with wavelengths different to that of the TESs. These peaks correspond to bulk modes which allow strong electric field distributed at the second to the ninth nanobeams (Fig. 2h). It is clearly that the FDTD results are in good agreement with our analytical tight binding analysis. The field strength of the TESs is determined by an exponential decay function $\left( {\frac{{\kappa_{2} }}{{\kappa_{1} }}} \right)^{ - n}$^42,44^, where $\kappa_{1}$ ($\kappa_{2}$) represents the intra (inter) coupling strength, $n$ ($n = 1, 2, \ldots N/2$) stands for the $n$-th nanobeam in the SSH structure, and $N$ denotes the total number of the nanobeams and is even integer. For example, when we increase the intra-coupling strength $\kappa_{1}$ by reducing $d_{1}$ from 0.8 to $0.7 \;{\upmu{\text{m}}}$ while keeping the inter-coupling strength $\kappa_{2}$ by keeping $d_{3} = 0.6 \;{\upmu{\text{m}}}$, as shown in Fig. S5 in Supplementary Information, the exponential decay function increases and thus the electric field decays slower from the edge to the middle nanobeams. It is noted from Fig. 2b,c that the first TES decays faster from edge nanobeams to middle nanobeams that that of the second TES, since it has a larger exponential decay function. In our FDTD simulations, multiple electric dipoles are placed randomly in each nanobeam to excite all the possible resonance modes. Meanwhile, several monitors are added randomly in each nanobeam to record the time signal to retrieve the averaged optical spectra.

### Tailoring TESs via tuning coupling strength both axially and transversely

**Figure 3:**
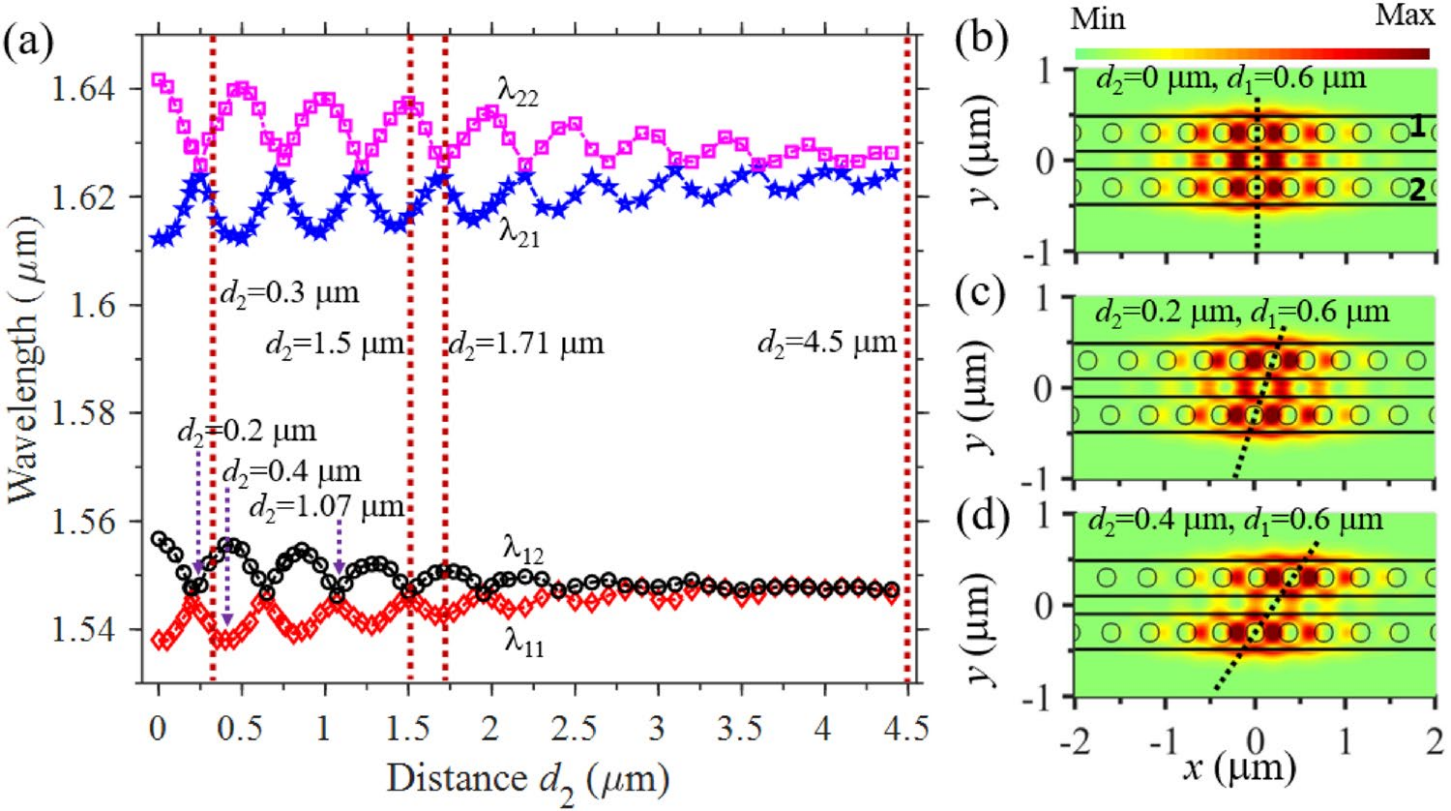
Influence of the axial shift $d_{2}$ to the wavelength splitting of the resonance modes of the double coupled-nanobeams (see Fig. 1a). (**a**) The dependence of the four splitting wavelengths (i.e., $\lambda_{11}$, $\lambda_{12}$, $\lambda_{21}$ and $\lambda_{22}$) on *d*_2_, which indicates quasi-periodic change of the coupling strength of the resonance modes and hence allows us to selectively generate TESs by the finite SSH nanobeams. (**b**–**d**) Zoom of the electric field distribution |*E*| in the middle plane of the nanobeam membrane (see the field distribution $H_{z}$ in Fig. S3 in Supplementary Information) at the splitting wavelengths of $\lambda_{11} = 1.538 \;{\upmu{\text{m}}}$ when $d_{2} = 0 \;{\upmu{\text{m}}}$, $\lambda_{11} = 1.546 \;{\upmu{\text{m}}}$ when $d_{2} = 0.2 \;{\upmu{\text{m}}}$, and $\lambda_{11} = 1.538 \;{\upmu{\text{m}}}$ when $d_{2} = 0.4 \;{\upmu{\text{m}}}$, respectively, where the black dotted lines link the central holes of the two nanobeams. The transverse spacing in above simulations is fixed at $d_{1} = 0.6 \;{\upmu{\text{m}}}$ and the other geometrical parameters are the same as those in Fig. 1.

Having established the underlying concept of the topological SSH nanobeams by alternatively varying the transverse spacing between the adjacent nanobeams, we continue to explore another interesting aspect of selectively manipulating the TESs by controlling the axial shift of the successive nanobeams. We investigate the wavelength splitting of the resonance modes of the two coupled nanobeams when their axial shift $d_{2}$ changes at a fixed transverse spacing $d_{1}$ (see Fig. 1a). Figure 3a demonstrates that the four splitting wavelengths vary quasi-periodically with $d_{2}$, which differ significantly from the wavelength splitting behavior in Fig. 1e. We observe that when $d_{2}$ changes from 0 to 0.2 μm, the splitting wavelengths of $\lambda_{11}$ ($\lambda_{21}$) move closer to its counterparts of $\lambda_{12}$ ($\lambda_{22}$), meaning the coupling strength of the two resonance modes supported by the two nanobeams becomes weaker. We can get insight into this optical property by looking at the field distributions at these splitting wavelengths. The air holes in the center of the two nanobeams have no axial shift to each other when $d_{2} = 0 \;{\upmu{\text{m}}}$ (Fig. 3b), and maximum optical coupling and maximal wavelength splitting occur (Fig. 3a). When $d_{2}$ increases to 0.2 μm, however, the air holes in the center of the two nanobeams are shifted by nearly half pitch to each other (Fig. 3c), resulting in small optical coupling and smaller wavelength splitting. When $d_{2}$ is further enlarged to 0.4 μm (Fig. 3d), the air holes in the cavity center of the two nanobeams are shifted by nearly one pitch to each other, and the mode coupling and wavelength splitting becomes stronger again.

Another intriguing feature is that the wavelength splitting of the first resonance mode (i.e., $\lambda_{11}$ and $\lambda_{12}$) and the second resonance mode (i.e., $\lambda_{21}$ and $\lambda_{22}$) respond differently to the change of $d_{2}$ due to their different field distributions. Since the first resonance mode has smaller mode volume and hence smaller field spreading in the axial direction, its mode coupling tends to change faster when $d_{2}$ varies. It is found that the wavelength splitting of the first resonance mode is smaller than that of the second resonance mode when $d_{2} = 1.5 \;{\upmu{\text{m}}}$, but becomes larger than that of the second resonance mode when $d_{2} = 1.71 \;{\upmu{\text{m}}}$ as depicted by the vertical dotted lines in Fig. 3a. This unique feature allows us to selectively enable TESs by the finite SSH nanobeams. When the axial shift is as large as $d_{2} = 4.5 \;{\upmu{\text{m}}}$, the resonance modes in the two nanobeams are separated far away from each other and consequently mode coupling no longer occurs.

**Figure 4:**
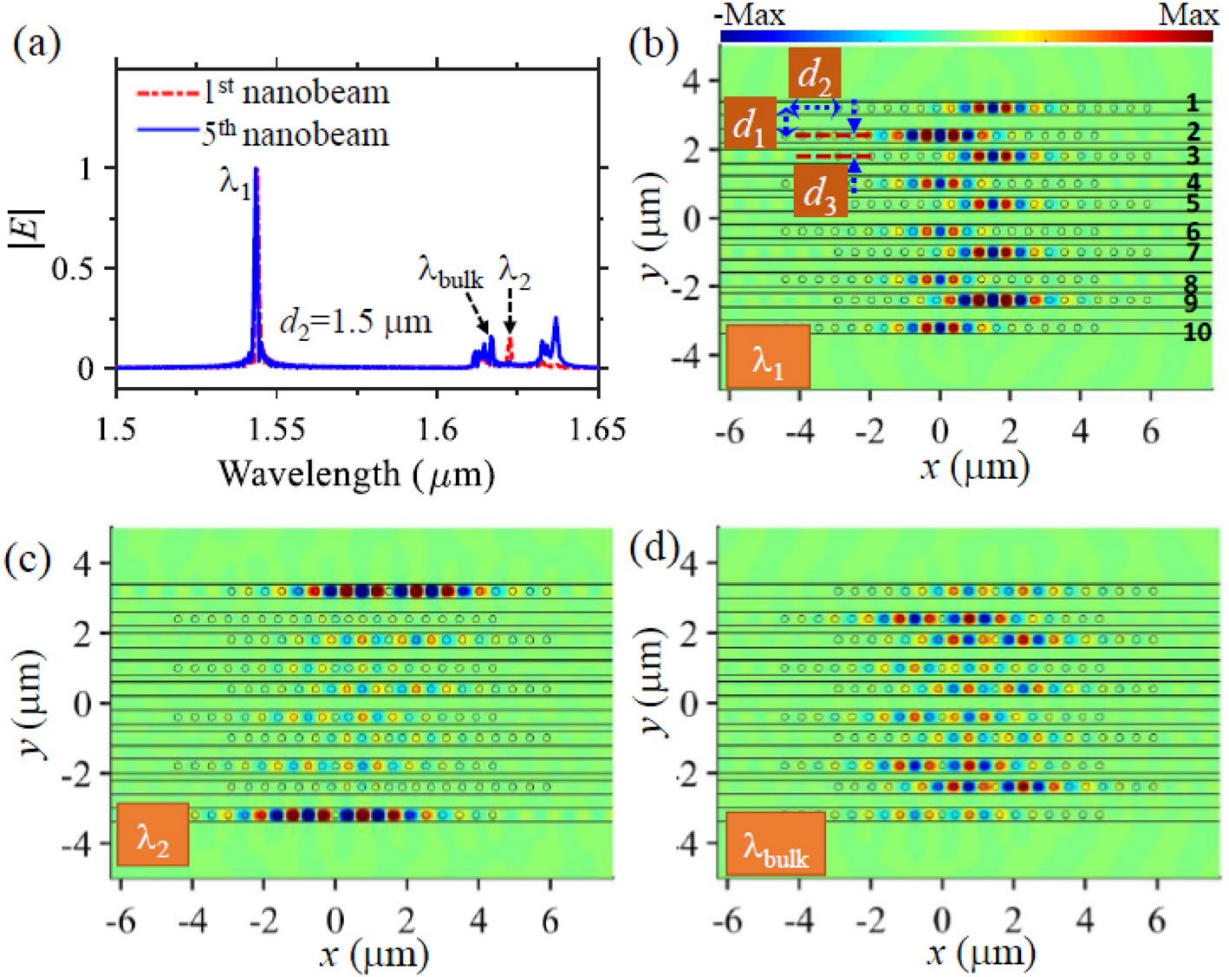
Optical properties of the TESs enabled by SSH configuration with axial shift of $d_{2} = 1.5 \;{\upmu{\text{m}}}$ between the adjacent nanobeams. (**a**) The spectra of the first and the fifth nanobeams of the SSH structure, showing a merged peak at wavelength $\lambda_{1 } = 1.546\;\upmu{\text{m}}$ and suggesting the edge mode not being allowed at $\lambda_{1 }$ due to weak coupling strength at this wavelength. (**b**–**d**) The real part of the field distribution $H_{z}$ of the SSH structure in the middle plane of the nanobeam membrane at $\lambda_{1 } = 1.546\;\upmu{\text{m}}$, $\lambda_{2 } = 1.624\;\upmu{\text{m}}$, $\lambda_{\text{bulk}} = 1.617\;\upmu{\text{m}}$, respectively. The geometrical parameters of the SHH structures are the same as those in Fig. 2 except a $1.5 \;{\upmu{\text{m}}}$ axial shift between the neighboring nanobeams.

**Figure 5:**
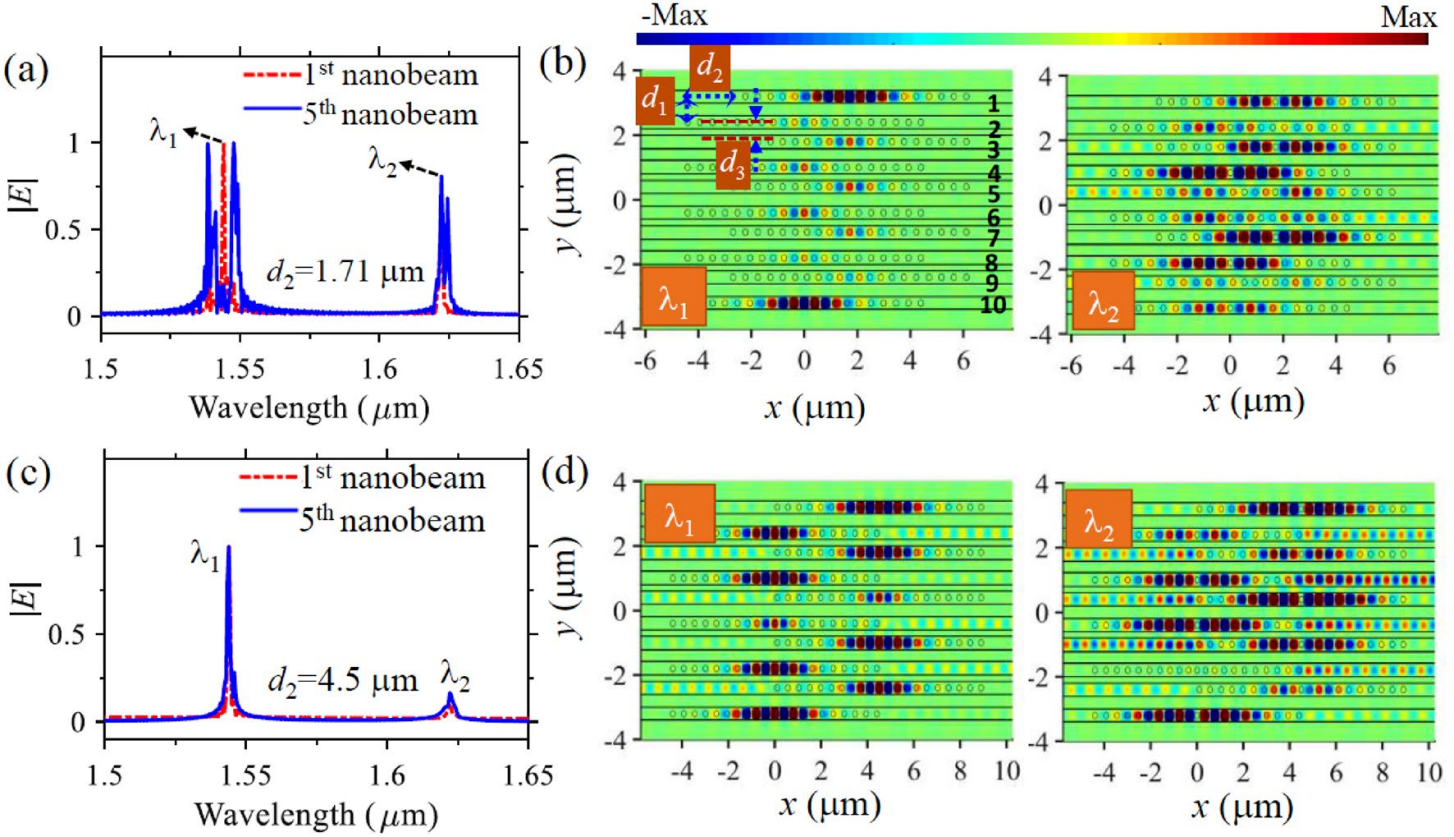
Tailoring TESs with different axial shift of the SSH nanobeams. (**a**) Spectra of the edge and middle nanobeams of the SSH structure when $d_{2 } = 1.71\;\upmu{\text{m}}$, showing a merged peak at $\lambda_{2 } = 1.624\;\upmu{\text{m}}$ and suggesting the edge mode not being allowed at this wavelength. (**b**) The corresponding electric field distribution |*E*| at the spectra peak wavelengths $\lambda_{1}$ (left) and $\lambda_{2}$ (right), respectively. (**c**) Spectra of the edge and middle nanobeams of the SSH structure when $d_{2 } = 4.5\;\upmu{\text{m}}$, showing merged peaks at both $\lambda_{1 } = 1.546\;\upmu{\text{m}}$ and $\lambda_{2 } = 1.624\;\upmu{\text{m}}$ and indicating non generation any edge mode. (**d**) The corresponding field distribution $H_{z}$ at the two wavelengths. The other geometrical parameters of the nanobeams are the same as those in Fig. 4.

Given the fact that the coupling strength of the resonance modes depends on the axial shift of the adjacent nanobeams as just revealed, another degree of freedom can be added to tailor TESs. It is possible to selectively generate either of the TESs by carefully shifting the nanobeams axially. We observe from Fig. 4 that the resonant mode at $\lambda_{1 } = 1.546\;\upmu{\text{m}}$ is no longer an edge mode when the axial shift changes from $d_{2 } = 0\;\upmu{\text{m}}$ to $d_{2 } = 1.5\;\upmu{\text{m}}$. In this scenario, the first resonance mode has low coupling strength (Fig. 3a) and does not support strong alternative intra and inter coupling between the adjacent nanobeams even though the structure has SSH configuration. As a result, the resonant mode at $\lambda_{1}$ is excited in each nanobeam cavity, and no edge states appear for $\lambda_{1}$ as indicated by the optical spectrum (Fig. 4a), where both the edge and middle nanobeams share the same spectra peak wavelength at $\lambda_{1}$. This is evidenced by the field profile plotted in Fig. 4b, where the electric field is distributed along all the ten nanobeams. When $d_{2 } = 1.5\;\upmu{\text{m}}$, TES appears only for the second resonance mode at $\lambda_{2 } = 1.624\;\upmu{\text{m}}$ because of strong light coupling between the neighboring nanobeams only at this wavelength. The edge nanobeams (i.e., the first and tenth nanobeams) have a spectral peak at $\lambda_{2 }$, and support strong localized electric field along them (Fig. 4c). The field distribution of the bulk mode at $\lambda_{\text{bulk}} = 1.615\;\upmu{\text{m}}$ is plotted in Fig. 4d. We see that the bulk mode has electric field mainly distributed at second to ninth nanobeams, which differs to the field profiles of both the resonant mode at $\lambda_{1}$ and the edge mode at $\lambda_{2 } .$ Based on the same concept, we can select the first resonance mode to become TES and at the same time disable the topological edge state property of the second resonance mode by changing the axial shift to $d_{2 } = 1.71\;\upmu{\text{m}}$ (Fig. 5a,b), since in this case the first resonance mode supports high coupling strength while the second resonance mode has small coupling strength as suggested by Fig. 3a. Since the proposed SSH nanobeams allow topological edge modes with high Q factor and small mode volume, they could find promising application of topological lasers. Four-level two-electron model can be utilized to investigate the topological lasing dynamics including electron populations of the levels, pumping threshold, and lasing spectrum^10^. Topological insulator lasers based on one-dimensional SSH micro-ring resonators have been recently demonstrated^42,43^. It is well known that micro-ring resonators generate multiple resonant modes, hence these SSH micro-ring resonators introduce multiple TESs and the operating wavelength of these lasers may not be stable and could drift from one TES to the other due to the fluctuation of the pumping condition (such as pumping power and position instability). Since the proposed concept can support single TES by carefully controlling transverse and axial shift of the SSH nanobeams, it could find potential application of stable single-frequency topological insulator laser generation. When the axial shift is as large as $d_{2 } = 4.5\;\upmu{\text{m}}$, both resonance modes have weak coupling strength and thus do not support edge modes, as demonstrated by the spectra in Fig. 5c and the field distribution in Fig. 5d.

**Figure 6:**
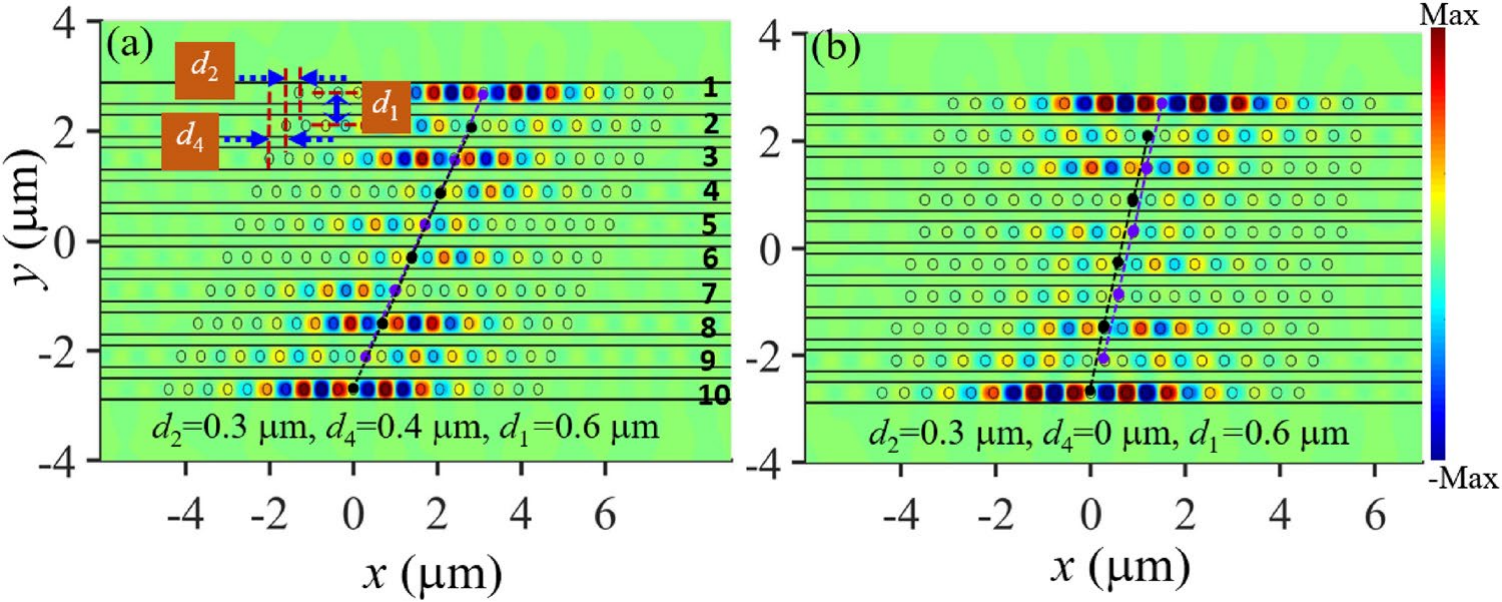
Generation of TESs by the SSH nanobeams with the same transverse spacing but different axial shift. (**a**) The field distribution $H_{z}$ of the edge mode at $\lambda_{2} = 1.624\;\upmu{\text{m}}$ when $d_{2 } = 0.3\;\upmu{\text{m}}$ and $d_{4 } = 0.4\;\upmu{\text{m}}.$ (**b**) The field distribution |*E*| of the edge mode at $\lambda_{2} = 1.624\;\upmu{\text{m}}$ when $d_{2 } = 0.3\;\upmu{\text{m}}$ and $d_{4 } = 0\;\upmu{\text{m}}$. In the SSH structures, all the nanobeams have the same transverse spacing of $d_{1 } = 0.6\;\upmu{\text{m}}$. The axial shift between the nanobeams $j$ to $j + 1$ ($j = 1, 3, 5, 7, 9$) is $d_{2 }$, and the axial shift between the nanobeams *j* to $j$ + 1 ($j$ = 2, 4, 6, 8) is $d_{4 }$, as depicted by the structure outline in (**a**). The other geometrical parameters of the nanobeams are the same as those in Fig. 5. The black and purple dotted lines indicate the central holes of the even and odd nanobeams, respectively. The spectra of the SSH nanobeams and the field distribution $\left| {\mathbf{E}} \right|$ of the generated edge mode at the first resonance mode wavelength $\lambda_{1}$ are given in Fig. S7 in Supplementary Information.

Lastly, we exploit another kind of SSH nanobeam structures to enable TESs using the same transverse spacing but different axial shift between the adjacent nanobeams. For example, two edge modes can be generated when the axial shift between the nanobeams *j* to $j + 1$ ($j = 1, 3, 5, 7, 9$) is $d_{2 } = 0.3\;\upmu{\text{m}}$, the axial shift between the nanobeams $j$ to $j + 1$ ($j = 2, 4, 6, 8, 10$) is $d_{4 } = 0.4\;\upmu{\text{m}}$, and all the nanobeams have the same transverse spacing of $d_{1 } = 0.6\;\upmu{\text{m}}$, as shown in Fig. 6a. This topological property is attributed to the mode coupling strength between the neighboring nanobeams being smaller at $d_{2 } = 0.3\;\upmu{\text{m}}$ than that at $d_{4 } = 0.4\;\upmu{\text{m}}$ under the same the axial shift $d_{1 }$(Fig. 3a), which guarantees the alternative intra and inter coupling required by the SSH model. Based on the same working principle, we can also enable TESs when $d_{2 } = 0.3\;\upmu{\text{m}}$ an $d_{4 } = 0\;\upmu{\text{m}}$. Figure 6a,b plot the electric field distribution of the generated edge mode at $\lambda_{2 } = 1.624\;\upmu{\text{m}}$.

## Discussion

To conclude, we have demonstrated a PTI strategy based on the finite SSH PhC nanobeam cavities in a semiconductor membrane. In contrast to the conventional one-dimensional photonic lattice SSH structures that the hopping amplitude decreases exponentially with the distance between the adjacent sites, the proposed SSH systems have hopping amplitude changing quasi-periodically with additional axial shift of the adjacent sites. This intriguing property allows for selective manipulation of TESs, which cannot be achieved by SSH structures with one-dimensional lattice. We have designed SSH nanobeams with various configurations, and have investigated the coupling strength, the spectral characteristics, and the field distributions of the TESs. The proposed structures can allow two edge states with different mode profiles in the telecommunication region. Moreover, we reveal that the two edge states can be flexibly controlled by tailoring the mode coupling strength. It is noted that a single edge state can be selectively chosen by carefully shifting nanobeams axially and transversely. We believe our approach open a new way to manipulate the coupling strengths in SSH models and provide and provide a new integrated photonic strategy to retrieve nontrivial topology.

## Supplementary Information

Supplementary Information.

## Data Availability

The data that support the findings of this study are available from the corresponding authors upon reasonable request.

## Code availability

All the computer codes that support the findings of this study are available from the corresponding authors upon reasonable request.
