# Supplementary material for: Tailoring topological edge states with photonic crystal nanobeam cavities
Source: Sci Rep. 2021 Jan 13;11:1055. doi: 10.1038/s41598-020-79915-6 (PMC7806710; doi:10.1038/s41598-020-79915-6)
Supplement: Supplementary file 1 — Supplementary Information. [file 41598_2020_79915_MOESM1_ESM.pdf]

Supplementary Information

Tailoring Topological Edge States with Photonic Crystal Nanobeam Cavities

*Yongkang Gong, Liang Guo, Stephan Wong, Anthony J. Bennett, and Sang Soon Oh**

*Email: OhS2@cardiff.ac.uk

A. Wavelength Splitting of the Two Coupled Nanobeam Cavities

The proposed single nanobeam cavity supports two high Q factor resonance modes in the telecommunication region. The first resonance mode has wavelength of $\lambda_s = 1.546$ µm with symmetrical H_z field distribution with respect to the $x = 0$ µm plane (Fig. S1a), and the second resonance mode is at wavelength of $\lambda_a = 1.624$ µm and has antisymmetric H_z field profile along the $x = 0$ µm plane (Fig. S1b). When the vertical spacing of the two coupled nanobeams is as large as $d_1 = 2$ µm, the two resonance modes have localized field at the center of each nanobeam and the modes from the two nanobeams do not couple to each other. When the spacing d_1 reducesto 0.6 µm, strong mode coupling occurs, and the two resonance modes

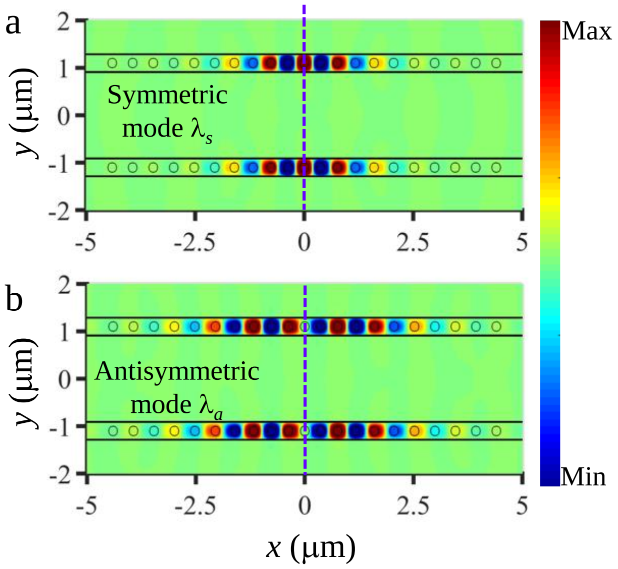

Fig. S1 The real part of the field distribution H_z of the symmetric mode at $\lambda_s = 1.546$ µm (a) and the antisymmetric mode at $\lambda_a = 1.624$ µm (b) in the plane of the central membrane (i.e., the $z = 0$ µm plane). The geometrical parameters of the nanobeams are the same as those in Fig. 1c in the main text. The purple dashed lines indicate the $x = 0$ µm plane.

are split (Fig. 1e in the main text). The first resonance mode is split into a mode at wavelength of $\lambda_{s,a}$ and a mode at wavelength of $\lambda_{s,s}$. The mode at $\lambda_{s,a}$ has symmetric (antisymmetric) H_z field profile with respect to the $x = 0$ µm ($y = 0$ µm) plane, while the mode at $\lambda_{s,s}$ has symmetric H_z field profile with respect to both the $x = 0$ µm and the $y = 0$ µm planes, as depicted in Fig. S2a-b. The second resonance mode is split into two modes at $\lambda_{a,a}$ and $\lambda_{a,s}$.

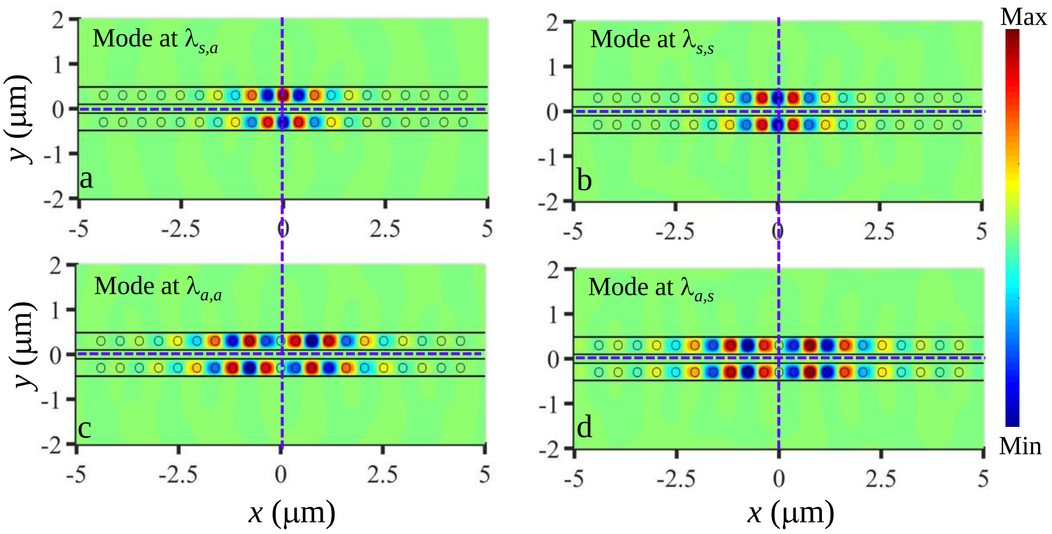

Fig. S2 The real part of the field distribution H_z of the splitting modes at $\lambda_{s,a}$ = 1.538 µm (a), $\lambda_{s,s}$ = 1.557 µm (b), $\lambda_{a,a}$ = 1.612 µm (c), and $\lambda_{a,s}$ = 1.642 µm (d), respectively. The geometrical parameters of the nanobeams are the same as those in Fig. 1d in the main text. The purple dashed lines indicate the $x = 0$ µm and the $y = 0$ µm planes.

Figure S2c-d indicates that the mode at $\lambda_{a,a}$ has antisymmetric H_z field profile with respect to both the $x = 0$ µm and the $y = 0$ µm planes, while the mode at $\lambda_{a,s}$ has antisymmetric (symmetric) H_z field profile with respect to the $x = 0$ µm ($y = 0$ µm) plane. The horizontal spacing d_2 between the two nanobeams affect the coupling strength of the resonance modes as well. The mode coupling strength changes quasi-periodically with d_2 (Fig. 3A in the main text). Figures 3c and 3d in the main text show the field distribution $|E|$ at the splitting wavelength of 1.546 µm when $d_2 = 0.2$ µm and at the splitting wavelength of 1.538 µm when $d_2 = 0.4$ µm. Figure S3 depicts their corresponding H_z field distribution.

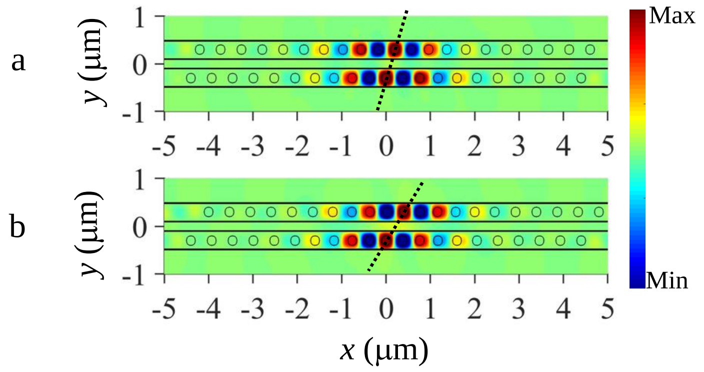

Fig. S3 The real part of the field distribution H_z of the two coupled nanobeams at the splitting wavelength of 1.546 μm when $d_2 = 0.2$ μm (a) and at the splitting wavelength of 1.538 μm when $d_2 = 0.4$ μm (b), respectively. The geometrical parameters of the nanobeams are the same as those in Fig. 3c-d in the main text. The black dotted lines link the center of the two nanobeams.

B. Tight Binding Analysis and Topological Nature

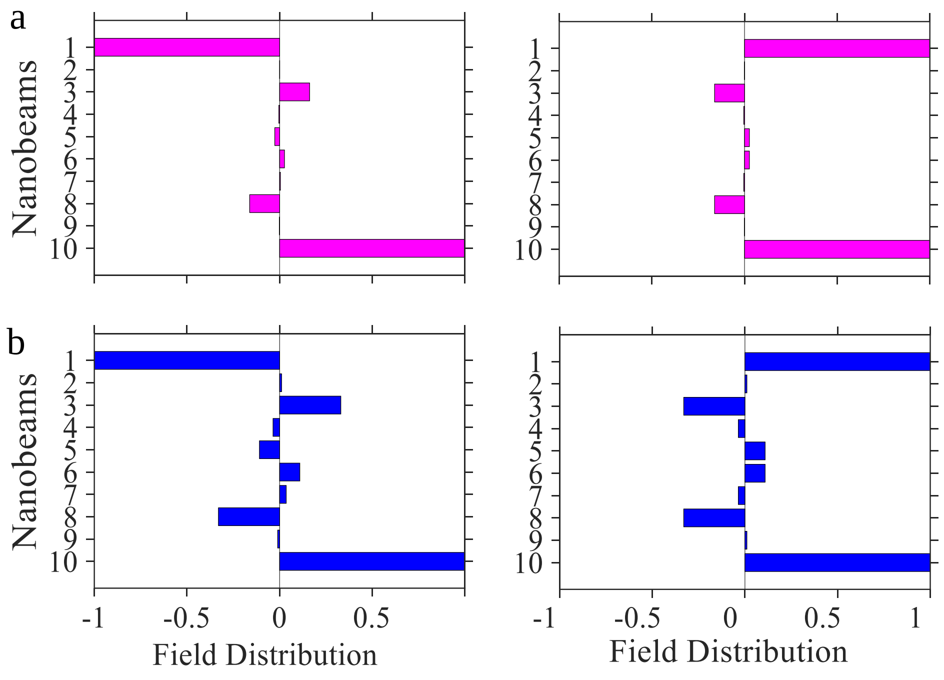

Fig. S4 The normalized field distribution (the real part of the eigenvectors) of the two degenerate zero-energy TESs at the first resonance wavelength λ_1 (**a**) and the second resonance wavelength λ_2 (**b**) obtained by the tight binding model. The simulation paraments are the same as those in Fig. 2a-c in the main text.

The proposed SSH structure has ten nanobeams with alternatively changed spacing between

the successive nanobeams, as indicated by structure outline in Fig. 2f in the main text. With the nearest-neighbor approximation, the Hamiltonian of the finite SSH nanobeams can be given by

$$H = \begin{pmatrix} 0 & \kappa_1 & & & \\ \kappa_1 & 0 & \kappa_2 & & \\ & \kappa_2 & \ddots & \kappa_1 & \\ & & \kappa_1 & 0 & \kappa_2 \\ & & & \kappa_2 & 0 \end{pmatrix}_{N\times N}, \tag{1}$$

where N is the total number of the nanobeams, and κ_1 (κ_2) is the intra-cell (inter-cell) coupling strength. The diagonal terms can be viewed as the detuning with respect to resonance frequency. By including the obtained coupling strength (see Fig. 1F in the main text) in above equation, we can derive the eigenvalues and the eigenvectors of the proposed SSH nanobeam structures. The field distribution of the two zero-energy TESs at the first and second resonance mode wavelengths have localized field at edge nanobeams in symmetric or antisymmetric manners, as shown in Fig. S4. Figure 2 in the main text and Fig. S5 demonstrate that our tight binding analysis agrees well with the FDTD results.

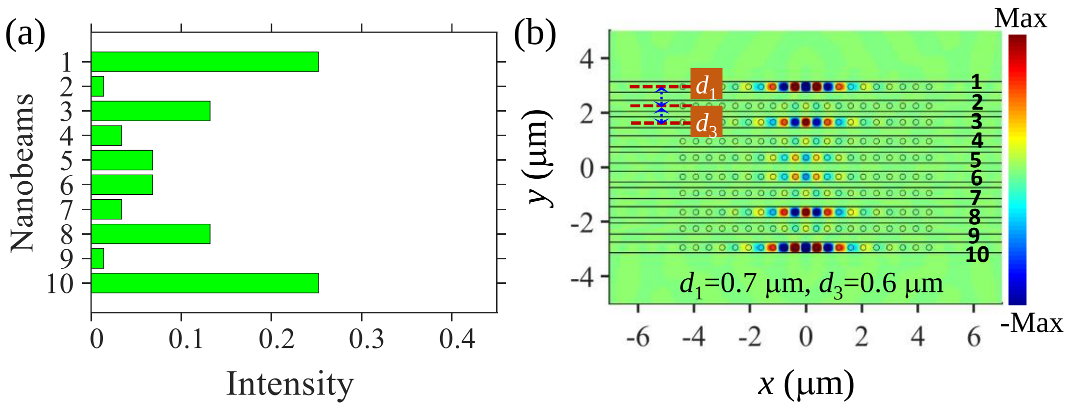

Fig. S5 The conventionally normalized intensity (**a**) and the field distribution H_z (**b**) of the first TES obtained by the tight bonding model and the FDTD method. Here, $d_1 = 0.7$ μm, $d_3 = 0.6$ μm, and the other geometrical parameters are the same as those in Fig. 2 in the main text.

C. Topological Robustness of The TESs

An important feature of the topological edge mode is its robustness against disorder. Disorder in the coupling strength and direct modification of the edge site hardly vary the properties of TESs in SSH photonic systesms[1-3]. We consider a local perturbation by introducing the random fluctuation to the intra-cell and inter-cell coupling, as illustrated in Fig. S6a. The Hamiltonian

of the perturbed SSH nanobeams is determined by

$$H = \begin{pmatrix} 0 & \kappa_1(1+\Delta\kappa_1) & & & \\ \kappa_1(1+\Delta\kappa_1) & 0 & \kappa_2(1+\Delta\kappa_2) & & \\ & \kappa_2(1+\Delta\kappa_2) & \ddots & \kappa_1(1+\Delta\kappa_9) & \\ & & \kappa_1(1+\Delta\kappa_9) & 0 & \kappa_2(1+\Delta\kappa_{10}) \\ & & & \kappa_2(1+\Delta\kappa_{10}) & 0 \end{pmatrix}_{N\times N}. \quad (2)$$

The eigenvalues of the Hamiltonian versus disorder factor are obtained and plotted in Figs. S6b and S6c, which demonstrated that the zero modes are robust against the disorder.

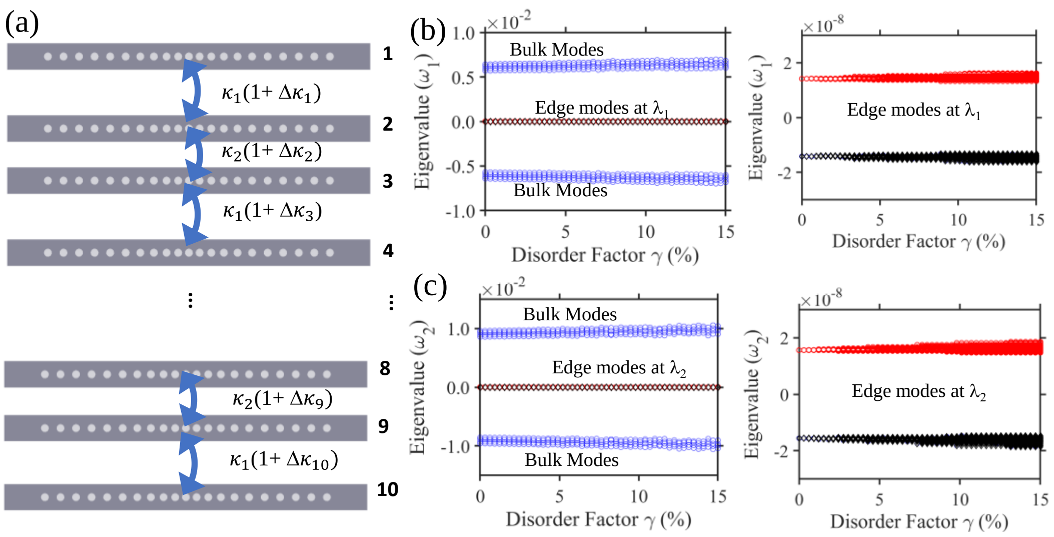

Fig. S6 (a) Schematics of the SSH nanobeams with random fluctuations introduced to the coupling strength of the neighboring nanobeams. The intra-cell and inter-cell coupling strength become $\kappa_1(1+\Delta\kappa_i)$ and $\kappa_2(1+\Delta\kappa_j)$, where $\Delta\kappa_{i(j)} = \gamma \times rand$, $i = 1, 3, \ldots, 9$, and $j = 2, 4, \ldots, 10$. γ is disorder factor and gives uniformly distributed random number in the interval (0,1). (b) The normalized eigenvalues of the Hamiltonian versus the disorder factor at the first resonance wavelength λ_1 (left) and the zoom-in zero modes (right). (c) The normalized eigenvalues of the Hamiltonian versus the disorder factor at the second resonance wavelength λ_2 (left) and the zoom-in zero modes (right).

D. Optical Properties of the SSH Nanobeams With the Same Vertical Spacing

Since mode coupling strengthen can be tuned by the horizontal shift of the nanobeams (Fig. 3a), we can tailor TESs with SSH structures having the same vertical spacing but different horizontal shift between the adjacent nanobeams, as demonstrated in Fig. 6 in the main text and Fig. S7.

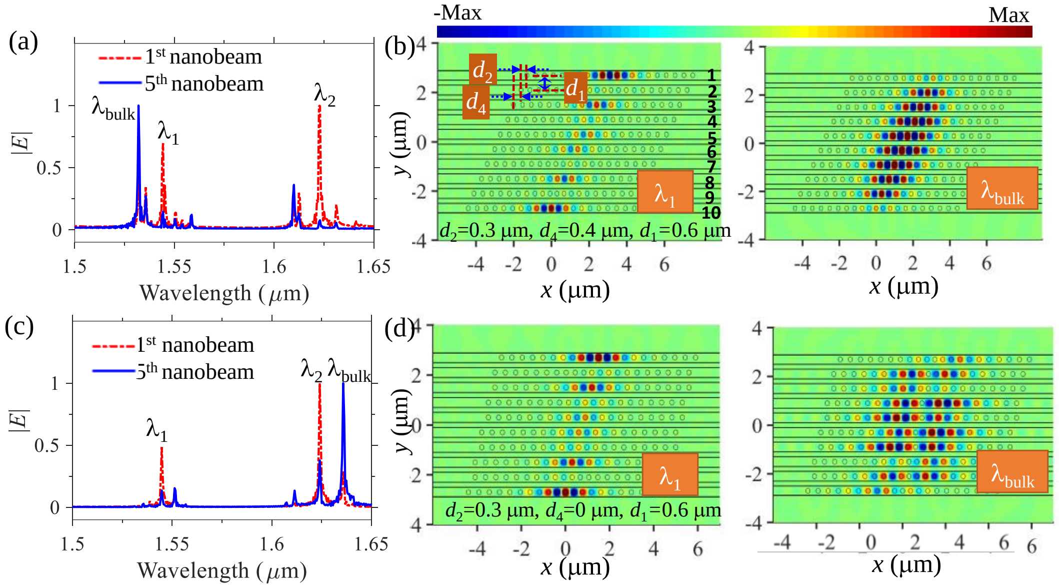

Fig. S7 (a) The optical spectra of the edge and middle nanobeams when $d_2 = 0.3$ μm and $d_4 = 0.4$ μm. (**b**) The corresponding field distribution H_z of the edge mode at $\lambda_1 = 1.546$ μm (left figure) and the bulk mode at $\lambda_{\text{bulk}} = 1.532$ μm (right figure). (**c**) The optical spectra of the edge and middle nanobeams when $d_2 = 0.3$ μm and $d_4 = 0$ μm. (**d**) The corresponding field distribution H_z of the edge mode at $\lambda_1 = 1.546$ μm (left figure) and the bulk mode at $\lambda_{\text{bulk}} = 1.636$ μm (right figure). All the adjacent nanobeams have the same vertical spacing of $d_1 = 0.6$ μm. The geometrical paraments of SSH structures are the same as those in Fig. 6 in the main text.

References

1. Zhao, H., *et al.* Topological hybrid silicon microlasers, *Nat. Commun.* **9**, 981 (2018).

2. Han, C., Lee M., Callard S., Seassal C. & Jeon H. Lasing at topological edge states in a photonic crystal L3 nanocavity dimer array, *Light Sci. Appl.* **8**, 40 (2019).

3. Pan, M., Zhao H., Miao P., Longhi S. & Feng L. Photonic zero mode in a non-Hermitian photonic lattice, *Nat. Commun.* **9**, 1308 (2018).
